# User Perceptions of a Dementia Risk Reduction Website and Its Promotion of Behavior Change

**DOI:** 10.2196/resprot.2372

**Published:** 2013-04-18

**Authors:** Maree Farrow

**Affiliations:** ^1^Alzheimer's AustraliaHawthornAustralia; ^2^Centre for Research on Ageing, Health and WellbeingThe Australian National UniversityCanberraAustralia

**Keywords:** dementia, Alzheimer's disease, risk reduction behavior, health communication, evaluation studies

## Abstract

**Background:**

Several modifiable health and lifestyle factors are consistently associated with dementia risk and it is estimated that significantly fewer people would develop dementia if the incidence of risk factors could be reduced. Despite this, Australians’ awareness of the health and lifestyle factors associated with dementia risk is low. Within a national community education campaign, Alzheimer’s Australia developed a dementia risk reduction website providing information about modifiable risk or protective factors for dementia.

**Objective:**

This study aimed to assess the usefulness of the website content in improving knowledge and enabling adoption of recommended strategies, and to examine what additional resources consumers need.

**Methods:**

Visitors to the website over a 3 month period were invited to complete an online survey, which asked them to rate their knowledge of dementia risk reduction before and after visiting the site, how important monitoring their health related behavior was to them before and after visiting the site, their current behavior related to health and lifestyle factors associated with dementia risk, their intentions to change behavior, and the usefulness of potential additional resources to help them do so.

**Results:**

For this study, 123 Australian adults responded to the survey. 44.7% (55/122) were aged over 60 and 82.1% (98/119) were female. Respondents’ ratings and comments indicated they generally found the content interesting, informative, and helpful to them. Respondents’ ratings of their knowledge about the links between health and lifestyle factors and dementia risk significantly increased after visiting the website (*P*<.001). Their ratings of how important monitoring what they do in relation to their health and lifestyle factors were also significantly increased after visiting the website (*P*<.001). Average ratings for how well respondents felt they were doing at the time in relation to specific risk or protective factors were generally high, suggesting many website visitors already had high levels of health motivation and healthy lifestyle behaviors. 55.6% (45/81) said that after visiting the website their intention to make lifestyle changes was strong. Only 27.1% (22/81) said their intention to visit their doctor to discuss dementia risk reduction was strong. Potential additional resources that would help people assess and address their personal dementia risk factors were rated as more helpful than general information resources.

**Conclusions:**

A dementia risk reduction website providing information about the current evidence and practical strategies was of interest and was useful to the Australian community. Benefits for visitors included increased knowledge and increased motivation to address relevant behaviors. Many visitors to the site were already health conscious, indicating that more needs to be done to get dementia risk reduction messages to the wider community. More interactive and personalized resources in future interventions may offer additional benefits to individuals.

## Introduction

### Background

The prevalence of dementia worldwide is increasing with the ageing population. In Australia, it is projected to increase four-fold to around 1 million people by 2050 [1], with significant impacts on the health system and economy. While dementia remains incurable, preventative health approaches to address dementia risk factors offer some hope of reducing this impact. Efforts to provide a preventative health strategy for dementia are increasingly seen as worthwhile, and vital to curbing the growing number of people who are affected [2-6].

Several modifiable health and lifestyle factors are consistently associated with the risk of developing dementia from all causes, including Alzheimer’s disease [7-9]. Midlife hypertension [10], midlife high total cholesterol [11], diabetes [12], midlife obesity [13], high saturated fat consumption [14], head injury [15], and smoking [16] are associated with greater risk of developing dementia. Odds ratios vary between studies and meta-analyses, but generally these factors are associated with 1.5-3 times increased risk of dementia. Regular physical exercise [17], mental and social activity [18,19], and higher fruit and vegetable and unsaturated fat consumption [14] are associated with reduced risk of developing dementia. These factors are typically associated with 30-70% reduced risk of dementia.

Preventative health approaches that facilitate lifelong mental, physical, and social activity, healthy eating and lifestyles, and prevention or control of vascular risk factors have the potential to reduce the number of people developing dementia [9]. Barnes and Yaffe recently estimated that 3 million cases of Alzheimer’s disease could be prevented worldwide by reducing by 25% the incidence of 7 risk factors (diabetes, midlife hypertension, midlife obesity, depression, physical inactivity, smoking, and cognitive inactivity, [2]). Computer modelling based on population growth estimates and dementia prevalence data showed that significant impacts could be achieved by modifying the risk factor profile in the Australian population. For example, a decline in the physical inactivity rate by 5% every 5 years would reduce dementia prevalence by 11% in 2051 [20].

Despite this potential, most people have little knowledge about dementia risk factors [21]. A 2008 review of surveys revealed that, on average, 51% of Australians believed risk reduction is possible, while 20% believed nothing can be done to reduce dementia risk, and the remainder were unsure [21]. When asked how risk could be reduced, or when presented with possible reduction factors and asked which would reduce risk, mental activity was nominated by more Australians than any other strategy, followed by a healthy diet and physical exercise. The majority of people did not agree that reducing vascular risk factors (smoking, high blood pressure, and high cholesterol) could reduce dementia risk, highlighting a pressing need to educate the public that preventing or managing vascular risk factors can reduce risk of developing dementia in addition to heart disease and stroke [21].

### Mind Your Mind

To improve public awareness, Alzheimer’s Australia (Australia’s national dementia association) developed a community education program about dementia risk reduction called Mind your Mind (MYM). The program provides information about health and lifestyle behaviors associated with lower risk of developing dementia. The 7 MYM “signposts” (body, brain, diet, habits, head, health checks, social life) deal with aspects of behavior related to modifiable risk factors for dementia. The program initially relied on community education forums and printed resources, but these have limited reach. To improve accessibility to MYM, the program now includes online resources as described in the Methods section below.

In developed countries including Australia, the vast majority of people are Internet users (in 2011 an estimated 89.8% of Australians were Internet users [22]). The use of the Internet for health information is increasing, in part because people like the convenience and anonymity it provides [23-25]. Web-based health information therefore has the potential to reach large audiences at low cost [26,27]. Web-based interventions are also found to be effective at increasing awareness and enabling healthy behavior changes. Many studies report positive changes in knowledge, attitude, awareness, and healthy behavior for participants using Web-based health interventions, suggesting that Web-based health resources are capable of promoting healthy lifestyles [28,29].

In 2010, a MYM website was launched [30]. By assessing user perceptions of this website, this study aimed to determine whether evidence-based advice provided online is effective in promoting the uptake of dementia risk reduction approaches by individuals. Specifically, the study aimed to evaluate the effectiveness of the information provided in improving knowledge about dementia risk factors and the lifestyle and health strategies that may reduce risk, the effectiveness of the information provided in motivating and enabling individuals to adopt healthy behaviors, and what additional resources might assist individuals in adopting dementia risk reduction behaviors.

## Methods

### The MYM Website

**Figure 1:**
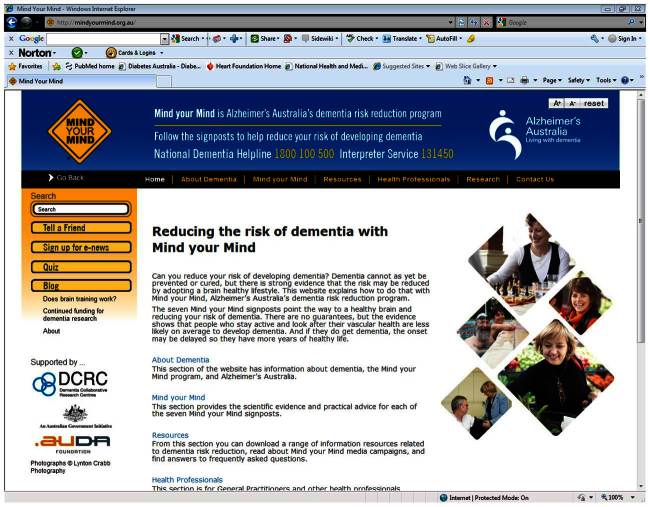
Screenshot of the Mind your Mind website home page.

The MYM website was developed to provide the Australian community with accessible and engaging information about the current evidence for lifestyle and medical factors associated with dementia risk. Figure 1 shows a screenshot of the MYM website home page. Table 1 describes the website sections and their contents at the time of this study. The main section of the website described the modifiable risk or protective factors associated with dementia, grouped under the 7 MYM signposts. For each factor, the current state of evidence was described in lay language and the strength of the evidence was rated using a 5-star system, and practical advice and links to relevant resources were provided. In developing the site content, Alzheimer’s Australia reviewed relevant literature, and used external experts’ advice on the recommendations being made regarding dementia risk reduction. The content was designed to be understood by Australian adults with average literacy and was not personalized for particular groups with differing levels of prior knowledge. Feedback from Alzheimer’s Australia staff and consumer advisors about the appropriateness of the content for the general public was incorporated into the final content design.

### The Survey

An online survey was developed using SurveyMonkey [31]. Feedback from Alzheimer’s Australia staff and consumer advisors was incorporated into the final survey design. At the commencement of the study, a brief notice and link were provided on the MYM website homepage to a dedicated internal page that provided detailed participant information and a link to the survey. Participants were instructed to read through the website before completing the survey, both on the information page on the website and on the survey itself, to minimize the chance of completing the survey without having viewed the website. The survey consisted of 48 items including demographics and questions about knowledge of dementia risk reduction before and after visiting the website, motivation to do something to reduce dementia risk before and after visiting the website, current behavior, intentions to change lifestyle behaviors to adopt risk reduction strategies, and what additional resources people feel they need to be able to improve their risk reduction behaviors.

Most items asked respondents to rate a specific attribute of the information provided on the website using a 5-point rating scale. Respondents were also able to enter additional comments. Where items asked respondents to rate attributes of the various sections of the website or of the information provided about specific risk or protective factors, respondents were instructed to select “not applicable” for any sections or factors they had not looked at. The survey also included open questions about what lifestyle changes respondents intended to make and what additional resources might help them.

### Participants

Users of the website from April to June, 2011, were invited to participate in the study via an information page on the MYM website with a link to the survey. Eligible participants included adults 18 years of age and over residing in Australia. Participants were anonymous but the survey asked for demographic information including age, gender, place of residence, medical status, education, and occupation. 123 people responded to the survey, however not everyone completed all questions.

### Data Analysis

For items rating attributes of the website using a 5-point scale, the mean rating across participants who provided a rating was calculated. Non-responders to individual questions were excluded from the analysis of those questions. *t* tests were conducted to examine differences in ratings between before and after visiting the website, for knowledge about the links between specific risk or protective factors and dementia, and for the importance of monitoring actions in relation to specific risk or protective factors. To correct for multiple comparisons, a significance level of *P*<.001 was applied.

**Table 1:** Description of the contents of each section of the MYM website.

Section	Contents
About dementia	what is dementia, symptoms, forms of dementia, risk factors
MYM	summary of evidence, practical advice, and links to resources for risk or protective factors: mental activity, diet, physical activity, social activity, blood pressure, cholesterol, diabetes, body weight, smoking, alcohol, head injury
Resources	downloadable information sheets, brochures and papers, frequently asked questions, quiz answers
Health professionals	downloadable guidelines and summary of evidence, resources for clinicians and patients for risk or protective factors: mental activity, diet, physical activity, social activity, blood pressure, cholesterol, diabetes, body weight, smoking, alcohol, head injury, depression
Research	dementia prevention research generally, current Australian research
Quizzes	three 10-question multiple choice quizzes on MYM, the brain, and music
Blog	brief articles on new research, publications, or resources

## Results

### Participant Demographics

The majority of respondents were 50-69 years of age (see Table 2). There were 98/119 (82.4%) female respondents and 86/120 (71.7%) lived in urban areas. There were 32/117 (27.0%) respondents born outside Australia and 28/121 (23.1%) reported speaking a language other than English at home. Some form of post-secondary education was completed by 76/121 (62.9%) respondents, and 39/121 (32.2 %) respondents were retired. Reported current or previous occupations were grouped according to the Australian and New Zealand Standard Classification of Occupations [32] and 57/118 (48.3%) respondents worked in management or professional roles. Chronic medical conditions affecting day-to-day function were reported by 10/120 (8.3%) respondents, but none had dementia.

There were no significant differences between responders and non-responders to each section of the survey regarding age group (*P*>.009), gender (*P*>.3), highest education level (*P*>.7), occupation classification (*P*>.3), or language spoken at home (English or non-English; *P*>.4).  Older respondents were more likely to drop out of the survey earlier and not complete all questions, but not significantly so.  The *P* values shown are representative of the lowest significance obtained from 3 *t* tests for each variable.  The variables were compared between responders and non-responders to the 3 sections of the survey that followed the first section on demographics.  For questions relating to the sections of the website, *P*=.03 for age, *P*=.66 for gender, *P*=.99 for education, *P*=.37 for occupation, and *P*=.43 for language.  For questions relating to the specific risk or protective factors, *P*=.009 for age, *P*=.52 for gender, *P*=.75 for education, *P*=.45 for occupation, and *P*=.91 for language.  For questions relating to potential additional resources, *P*=.01 for age, *P*=.37 for gender, *P*=.74 for education, *P*=.36 for occupation, and *P*=.99 for language.

**Table 2:** Characteristics of respondents.^a^

Characteristic		n (%)
**Age of respondents (N=122)**		
	18-30	10 (8.2)
	31-40	14 (11.5)
	41-50	12 (9.8)
	51-60	31 (25.4)
	61-70	40 (32.8)
	71-80	13 (10.7)
	>80	2 (1.6)
**Highest level of education completed by respondents (N=121)**		
	Year 6	2 (1.7)
	Year 8	3 (2.5)
	Year 10	23 (19.0)
	Year 12	17 (14.0)
	Diploma	25 (20.7)
	Bachelor degree	26 (21.5)
	Postgraduate degree	25 (20.7)
**Occupation classifications of respondents (N=118)**		
	Managers and self-employed	8 (6.8)
	Professionals	49 (41.5)
	Technicians and trade workers	5 (4.2)
	Community and personal service workers	12 (10.2)
	Clerical and administrative workers	31 (26.3)
	Sales workers	4 (3.4)
	Machinery operators and drivers	2 (1.7)
	Labourers	1 (0.8)
	Students and housewives	6 (5.1)

^a^N varied based on the number of respondents from 123 total participants who answered each question.

### Reasons for Interest in the Website

Respondents were asked what made them interested in dementia risk reduction and visiting the website. Table 3 shows the numbers of respondents who selected given reasons. Being worried about their memory or thinking was the most selected reason. Respondents were also asked what they hoped to learn from the website. Table 4 shows the numbers of respondents who selected given topics. What to do to reduce dementia risk was the most selected topic.

### Impressions of the Website and Content

Respondents overall impressions of the website were generally positive. Comments indicated that people found the content interesting, useful, and enlightening. For example, a female respondent past 70 years of age wrote,

Thank you for providing such a wealth of information on the subject. To have so much info on one site is fantastic.

However, a few respondents commented that the layout of the site could be made more interesting or that there was too much text. As shown in Table 5, the average rating of interest and appeal for most sections was between 4 (quite interesting) and 5 (very interesting), with a mean rating across sections of 4.07 (SD 0.33). The “MYM” section was rated the highest and the “blog” the lowest.

Respondents found the website easy to navigate. As shown in Table 5, average ratings for ease of navigation for each section were between 4 (somewhat easy) and 5 (very easy), with an average of 4.28 (SD 0.05) across sections. Comments about the navigation suggested that some people, while not finding it difficult, found using the drop down menus to navigate to another page annoying.

Respondents generally reported that the information provided was helpful to them, with ratings for most sections between 4 (quite helpful) and 5 (very helpful), as shown in Table 5, and a mean rating of 4.05 (SD 0.25) across sections. The “about dementia” section was rated the highest and the “blog” the lowest. Two people who felt they already knew a lot about dementia through having affected family members said that the website was not so helpful to them.

Respondents generally found the information provided easy to read and understand. As shown in Table 5, ease of understanding of website sections was rated between 3 (just right) and 4 (somewhat simplistic), with a mean rating across sections of 3.34 (SD 0.07). As shown in Table 6, ease of understanding of the information provided on specific risk or protective factors was also rated between 3 and 4, with an average rating across factors of 3.39 (SD 0.02). The information on alcohol was rated the closest to “just right” at 3.35. Comments indicated a high degree of satisfaction with understanding the content provided. For example, a female respondent in her 70s wrote, “The website is very good to read and understand specially for the people with English as a second language [*sic*]”. However, a male respondent in his 80s felt “the pertinent information is scattered around and not easily readable”.

### Impact on Dementia Risk Reduction Knowledge

**Figure 2:**
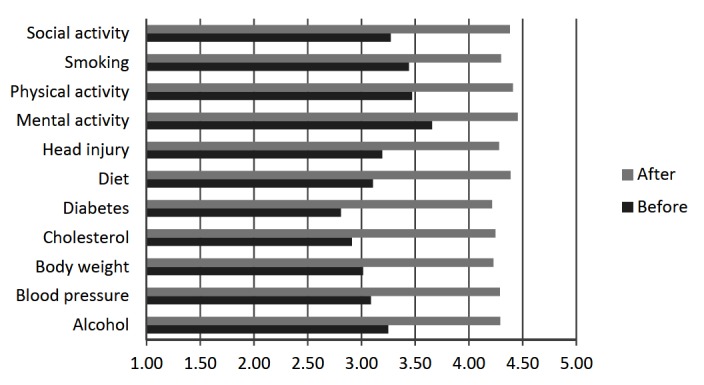
Mean ratings of knowledge about the link between risk or protective factors and dementia risk before and after visiting the Mind your Mind website (n=68). Ratings: 1=nothing at all; 2=not much; 3=a little; 4=quite a bit; 5=a lot.

As shown in Figure 2, average ratings of how much respondents knew about the links between specific risk or protective factors and dementia risk before reading the website were between 3 (a little) and 4 (quite a bit) for most factors. The average rating across factors was 3.20 (SD 0.25). Respondents rated their prior knowledge as lowest for diabetes (mean 2.81, SD 1.11) and highest for mental activity (mean 3.66, SD 0.90). Also shown in Figure 2, average ratings of how much respondents knew about the links between specific factors and dementia risk after reading the website were between 4 (quite a bit) and 5 (a lot), with an average rating across factors of 4.32 (SD 0.08). The largest improvement in knowledge was for diabetes (increase in mean rating of 1.41) and the smallest was for mental activity (increase in mean rating of 0.80). All factors showed significantly improved knowledge (*P*<.001) and the average increase in rating across factors was 1.12 (SD 0.02).

On average, respondents rated how much they learned from the website sections between 3 (something) and 4 (a fair bit), as shown in Table 5, with an average rating across sections of 3.75 (SD 0.17). Learning was rated highest for the “Research” section and lowest for the “Blog”. Some respondents commented that they had previous knowledge of the topics and so personally did not learn a lot. One female respondent in her 70s commented that she felt “someone with not much knowledge about dementia would learn a fair amount about it”, while a male respondent in his 60s felt that “most people will understand that they are actions which should be taken in any case for overall wellbeing”.

**Table 3:** Proportion of respondents who selected the given reasons for their interest in the website (N=116).

Reason	n (%)
I care for someone who has dementia	15 (12.9)
I have a family history of dementia	31 (26.7)
I feel I am getting to the age when dementia could affect me	34 (29.3)
I am worried about my memory or thinking	36 (31.0)
I am worried about someone close to me	22 (19.0)
The website was recommended to me by someone else	33 (28.4)
The information is relevant for my work	12 (10.3)

**Table 4:** Proportion of respondents who selected the given options for what they hoped to learn from the website (N=116).

Topic	n (%)
How to slow the progress of dementia	61 (52.6)
Whether dementia can be prevented	46 (39.7)
Whether I am at risk of getting dementia	51 (44.0)
Whether someone close to me is at risk of getting dementia	21 (18.1)
How to improve my memory or thinking	72 (62.1)
What to do to reduce the risk of getting dementia	90 (77.6)

**Table 5:** Mean (SD) ratings for questions asked in relation to website sections.

Section	Overall impression^a^	Easy to understand^b^	Easy to navigate^c^	Helpful information^d^	How much did you learn?^e^
	n=88 mean (SD)	n=86 mean (SD)	n=85 mean (SD)	n=83 mean (SD)	n=84 mean (SD)
Whole website	N/A	N/A	4.23 (0.91)	4.13 (0.82)	3.72 (0.96)
Home page	4.02 (0.78)	3.35 (0.74)	N/A	N/A	N/A
About dementia	4.38 (0.81)	3.36 (0.74)	4.26 (0.86)	4.34 (0.75)	3.81 (0.94)
MYM	4.51 (0.70)	3.33 (0.77)	4.28 (0.85)	4.26 (0.82)	3.84 (0.99)
Resources	4.11 (0.90)	3.26 (0.71)	4.33 (0.81)	4.10 (0.86)	3.83 (0.95)
Health professionals	3.85 (0.92)	3.31 (0.76)	4.26 (0.83)	3.89 (0.98)	3.71 (0.92)
Research	4.21 (0.99)	3.29 (0.80)	4.34 (0.75)	4.05 (0.94)	3.93 (0.91)
Quizzes	4.09 (1.02)	N/A	N/A	N/A	N/A
Blog	3.43 (1.38)	3.47 (0.73)	N/A	3.59 (1.10)	3.41 (1.08)

^a^What is your overall impression of the various sections of the website; how interesting and appealing are they? 1=not at all; 2=not very; 3=somewhat; 4=quite; 5=very (interesting)

^b^Is the information provided in the various sections of the website easy to read and to understand? 1=very complex; 2=somewhat complex; 3=just right; 4=somewhat simplistic; 5=very simplistic

^c^Do you find the website and its sections easy to navigate? 1=very difficult; 2=somewhat difficult; 3=neither easy nor difficult; 4=somewhat easy; 5=very easy

^d^How helpful is the information provided on the MYM website to you? 1=not at all; 2=not very; 3=somewhat; 4=quite; 5=very (helpful)

^e^How much do you feel you learned from the MYM website? 1=nothing at all; 2=not very much; 3=something; 4=a fair bit; 5=a great deal

**Table 6:** Mean (SD) ratings for questions asked in relation to specific risk or protective factors.

Risk /protective factor	Current behavior^a^	Intention to change^b^	Was information easy to understand?^c^	How well did information equip you?^d^	Are practical tips relevant and useful?^e^
	n=67 mean (SD)	n=67 mean (SD)	n=66 mean (SD)	n=65 mean (SD)	n=65 mean (SD)
Alcohol	4.16 (0.86)	3.75 (1.05)	3.35 (0.73)	4.06 (0.76)	4.03 (1.0)
Blood pressure	4.21 (0.73)	4.08 (1.04)	3.37 (0.78)	3.92 (0.91)	4.20 (0.86)
Body weight	3.77 (0.97)	4.23 (0.86)	3.39 (0.77)	4.00 (0.90)	4.19 (0.77)
Cholesterol	3.85 (0.78)	4.14 (0.98)	3.40 (0.77)	4.11 (0.86)	4.21 (0.86)
Diabetes /blood sugar	4.17 (0.80)	4.03 (1.07)	3.41 (0.79)	4.17 (0.81)	4.15 (0.86)
Diet	3.83 (0.84)	4.18 (0.99)	3.38 (0.76)	4.08 (0.9)	4.20 (0.75)
Head injury	4.38 (0.68)	4.29 (0.76)	3.38 (0.76)	4.10 (0.84)	3.96 (1.02)
Mental activity	4.20 (0.78)	4.26 (0.96)	3.39 (0.78)	4.05 (0.90)	4.31 (0.81)
Physical activity	3.64 (0.95)	4.36 (0.82)	3.41 (0.78)	4.11 (0.87)	4.18 (0.79)
Smoking	4.74 (0.59)	4.47 (0.72)	3.40 (0.77)	3.90 (1.04)	3.63 (1.33)
Social activity	4.02 (0.83)	4.23 (0.83)	3.39 (0.78)	4.05 (0.82)	4.20 (0.86)

^a^How well do you think you are currently doing in relation to the factors listed? 1=very badly; 2=somewhat badly; 3=could do better; 4=pretty well; 5=very well

^b^How strong is your intention to make changes to improve what you do in relation to the factors listed? 1=very weak; 2=somewhat weak; 3=considering it; 4=somewhat strong; 5=very strong

^c^How easy to understand was the information provided for each of the factors listed? 1=very complex; 2=somewhat complex; 3=just right; 4=somewhat simplistic; 5=very simplistic

^d^How well did the information provided equip you to improve what you do in relation to the factors listed? 1=not at all; 2=not much; 3=a little; 4=quite well; 5=very well

^e^Are the practical tips, activities, strategies and resources provided for the factors listed generally relevant and useful to you? 1=completely useless; 2=somewhat useless; 3=a little useful; 4=somewhat useful; 5=very useful

### Impact on Dementia Risk Reduction Importance

**Figure 3:**
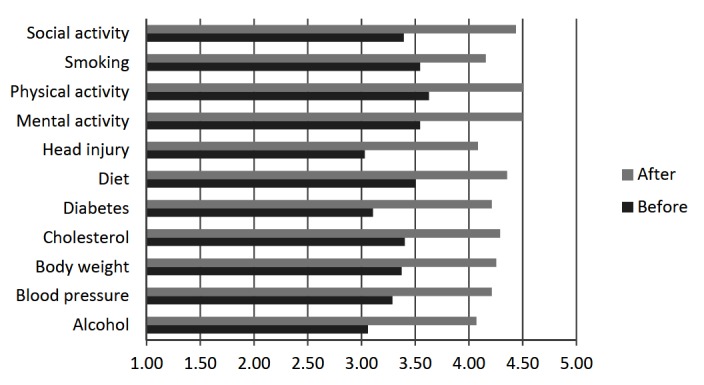
Mean ratings of importantance of monitoring behavior in relation to risk or protective factors before and after visiting the Mind your Mind website (n=67). Ratings: 1=not at all; 2=only mildly; 3=somewhat; 4=quite; 5=very (important).

As shown in Figure 3, average ratings of how important monitoring what they do in relation to specific risk or protective factors was to respondents before reading the website were between 3 (somewhat important) and 4 (quite important). The average rating across factors was 3.35 (SD 0.21). Respondents rated the importance of monitoring what they do as lowest for head injury (mean 3.03, SD 1.34) and highest for physical activity (mean 3.63, SD 1.22). Also shown in Figure 3, average ratings of how important monitoring what they do in relation to specific risk or protective factors was to respondents after reading the website were between 4 (quite important) and 5 (very important), with an average rating across factors of 4.28 (SD 0.15). The largest increase in rating of importance was for diabetes (increase in mean rating of 1.11) and the smallest was for smoking (increase in mean rating of 0.61). All factors except smoking (*P*=.02) showed significantly increased rating of importance (*P*<.001) and the average increase in rating across factors was 0.93 (SD 0.13). The information provided overall motivated 54/82 respondents (65.9%) to want to do something about reducing their dementia risk “quite a bit” or “a lot”. The mean rating for motivation was between “a little” and “quite a bit” (mean 3.66, SD 0.88).

### Practicality of the Information Provided

The average rating of how well respondents (n=81) felt the information provided equipped them to do something about reducing their risk of dementia was 3.78 (SD 0.89), between “a little” and “quite well”. 56 (69.1%) said the information provided equipped them “quite well” or “very well”. Average ratings of how well the information provided for specific risk or protective factors equipped respondents to improve what they do in relation to the factors are shown in Table 6. Mean ratings were between 4 (quite well) and 5 (very well) for most factors, with an average rating across factors of 4.05 (SD 0.08). The lowest average rating was for smoking and the highest was for diabetes.

As shown in Table 6, respondents rated whether the practical tips, activities, strategies, and resources provided for specific risk or protective factors were generally relevant and useful to them between 4 (somewhat useful) and 5 (very useful) for most factors. The average rating across factors was 4.12 (SD 0.19). The usefulness of the tips for mental activity was rated highest and that for smoking was rated lowest.

### Current Behavior

Average ratings for how well respondents felt they were currently doing in relation to specific risk or protective factors were between 3 (could do better) and 4 (pretty well) or 4 and 5 (very well), as shown in Table 6, with an average rating across factors of 4.09 (SD 0.31). Current behavior was rated highest for smoking and lowest for physical activity.

### Intention to Change Behavior

Of the 81 respondents, 45 (55.6%) rated their intention to make lifestyle changes to reduce their risk of dementia as 4 (somewhat strong) or 5 (very strong), and 28 (34.6%) rated their intention as 3 (considering it). The mean rating was 3.68 (SD 1.01). Only 22 (27.1%) said their intention to visit their doctor to discuss dementia risk reduction was “somewhat strong” or “very strong”, and a 21 (25.9%) were “considering it”. The mean rating was 2.63 (SD 1.29). Of those who felt they needed to change, respondents on average rated their intention to make changes to improve what they do in relation to specific risk or protective factors between 4 (somewhat strong) and 5 (very strong) for most factors, as shown in Table 6, with a mean rating across factors of 4.18 (SD 0.19). Intention to change was rated lowest for alcohol and highest for smoking.

**Figure 4:**
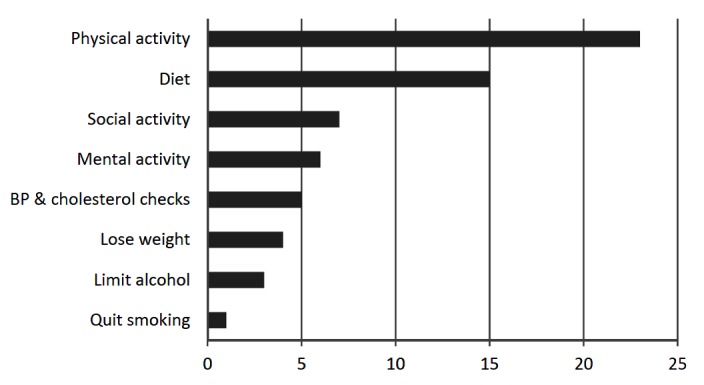
Number of respondents who stated intentions to make improvements in specific areas of behavior.

When asked to specify what changes they intend to make, 35 people provided a response. As shown in Figure 4, the most common intended change was to increase physical activity (23 respondents), followed by improving diet (15 respondents). Only one person said they intended to try to quit smoking, but among those for whom smoking was an issue, intention to change was rated very highly as stated above.

### Additional Resources Needed

Respondents rated how helpful they felt 12 suggested resources would be to them, if provided in addition to the information on the website. As shown in Table 7, average ratings were between 3 (somewhat helpful) and 4 (quite helpful) or 4 and 5 (very helpful). An online assessment to identify personal risk factors was rated highest and regular reminders to see a general practitioner was rated lowest. Several respondents commented that an online assessment to determine your individual percentage risk could be confronting, and that assessing and providing information about risk factors was a better approach. Some commented that a personalized dementia risk reduction program would need to involve their doctor. Others commented that regular reminders might be seen as “information overload”.

Respondents were asked an open question about what other information, advice, or resources they would like to see on the website and 18 people provided a response. Suggestions included opportunities to volunteer for research, information on local groups and clubs, diet and supplement information, personal stories, and information on other risk factors such as age and drugs. Respondents were also asked what else they needed to help them make changes in relation to specific risk or protective factors. The responses from 18 respondents are summarized in Table 8. Suggestions included fact sheets summarizing the important information and providing more tips to make it easier for people to take up the recommended strategies.

**Table 7:** Mean (SD) ratings of how useful respondents felt suggested additional resources would be to them (n=65). Ratings: 1=not at all; 2=not very; 3=somewhat; 4=quite; 5=very (helpful).

Potential resource	Mean rating (SD)
An assessment, that you could complete online, to determine your individual risk of developing dementia (eg, a 10% risk).	4.20 (1.01)
An assessment, that you could complete online, to identify your personal risk factors for developing dementia (that is, the things in your life that you could improve).	4.29 (0.80)
One off advice about what you can do to reduce your risk of dementia, based on your assessment, that is tailored to your individual circumstances.	4.19 (0.86)
Personalized dementia risk reduction programs for you to follow over time, based on your assessment and tailored to your individual circumstances.	4.10 (1.07)
Regular reminders and new challenges to help you follow your personalized dementia risk reduction programs.	4.03 (1.08)
The results of your assessment for you to take to your GP to help you discuss dementia risk reduction with them.	4.18 (0.98)
Regular reminders to see your GP to monitor any medical aspects of dementia risk reduction that apply to you.	3.76 (1.28)
General educational sessions about dementia risk reduction that you could attend to learn more, discuss issues and ask questions.	3.80 (1.16)
Activities or workshops to learn practical dementia risk reduction strategies (eg, healthy cooking, memory strategies, exercises).	3.81 (1.22)
Regular general updates, not personalized, such as a newsletter.	3.77 (1.05)
Regular updates on the latest dementia risk reduction research.	4.06 (0.97)
Information about services in your local area that you could utilize to help you do more dementia risk reduction activities.	4.06 (1.03)

**Table 8:** Selected responses to the open question “What else do you think you need to help you to make changes to improve what you do in relation to the factors listed? Are there any particular resources, facilities, information, services, etc, that you think would help you?”

Risk or protective factor	Suggested resources
Alcohol	Safe level of consumption Minimum consumption
Blood pressure	Fact sheet on lowering BP What range of BP is healthy
Cholesterol	List of foods with cholesterol ratings Checks by nurse at health center
Diet	List of foods with salt, sugar, etc ratings Recipes Diet plan
Mental activity	Brain exercises Information on different types of mental activity
Physical activity	Tips to make it easy to exercise
Social activity	Tips to make it easy to socialize

## Discussion

### Major Findings

This study aimed to evaluate users’ perceptions of a website providing information and resources related to health and lifestyle factors associated with the risk of developing dementia. An online survey revealed that the MYM website was viewed as being helpful and the majority of respondents perceived the website content to be interesting, informative, easy to understand, and useful.

The “MYM” and “About Dementia” sections were rated as the most interesting and helpful, suggesting visitors to the website were most interested in information about reducing dementia risk. This was supported by the finding that the most selected reasons given for visiting the website were concerns about memory or thinking and wanting to know what to do to reduce dementia risk. A recent Australian community survey found that the fear of developing dementia was second only to the fear of having cancer [33]. The fear increased with age, with 75% of people over 60 agreeing that they are afraid of developing dementia [33]. These findings suggest that there is much community interest in understanding what individuals can do to reduce their risk of dementia.

A study investigating the preferences of potential users of websites providing physical activity interventions found that ease of use was considered essential to the design of an appealing website [34]. Respondents found the MYM website easy to navigate and the content easy to understand. Ratings for how easy to understand the content was averaged between “just right” and “somewhat simplistic”, suggesting the site achieved the aim of providing complex information in simple, easy to understand user-friendly terms. Respondents also had no difficulty with navigation, suggesting the layout of information was appropriate to most users’ needs.

One of the principal aims of the MYM website was to improve knowledge about dementia risk factors and what individuals can do to reduce their risk. Respondents’ ratings of their knowledge about the links between given risk or protective factors and dementia risk significantly increased after visiting the website for all factors. Prior knowledge was rated highest for mental activity and physical activity, and lowest for diabetes and cholesterol. This was consistent with previous community survey findings that there was some awareness of the links between mental stimulation and dementia risk reduction, and physical health and dementia risk reduction, but there was very little awareness of the association with cardiovascular risk factors [21]. Encouragingly, knowledge about diabetes and cholesterol improved the most after visiting the website. Respondents’ subjective impression of how much they learned from the website was also encouraging, with an average rating approaching learned “a fair bit”.

Another principal aim of the MYM website was to promote behavior change by providing users with information and resources to assist them to adopt lifestyle and health strategies that may reduce their dementia risk. In this cross-sectional study, we did not measure actual behavior change, but assessed perceived importance of monitoring behavior and intentions to change behavior. Respondents’ ratings of how important to them monitoring what they do in relation to given risk or protective factors significantly increased after visiting the website for all factors except smoking. The smaller increase in importance for smoking was likely due to very few respondents being smokers (the highest average rating for how well respondents were currently doing in relation to risk or protective factors was for smoking).

The majority of respondents rated how much the information provided overall motivated them to do something about reducing their dementia risk as “quite a bit” or “a lot”, suggesting the website content was able to encourage users to consider behavior change. This was also supported by the finding that 73/81 (90.2%) respondents rated their intention to make lifestyle changes as “very strong”, “somewhat strong”, or “considering it’. Of course, intentions do not always translate into actions and it is well recognized that raising awareness and motivation are only part of the process of behavior change. Only 43/81 (53.1%) respondents rated their intention to visit their doctor as “very strong”, “somewhat strong”, or “considering it’. This may suggest that people do not see medical support in this area as important. The older age group making up the majority of respondents may already be having the recommended regular checks of cardiovascular risk factors, and therefore do not see the necessity to visit to the doctor another time.

Among those who felt they needed to change, the highest rating for intention to change was for smoking, followed by physical activity. There are many public messages about the dangers of smoking. Perhaps the association with increased dementia risk could help motivate smokers to try to quit. Physical activity is an issue many people think about given its prominence in public health messages and the media. More people specified increasing physical activity as a change they intended to make than any other factor.

The personal relevance and applicability of Web-based health information has previously been shown to be important to users [35,36]. On average, respondents rated whether the practical advice and resources provided for specific risk or protective factors were relevant and useful to them between “somewhat useful” and “very useful” for most factors, suggesting they did see the information as applicable to them as individuals. Mental activity was rated highest, suggesting users saw the tips for being more mentally active as particularly relevant to them. Smoking was rated lowest, likely due to few respondents being smokers. Respondents also on average rated the information provided in sections of the website to be helpful to them.

Potential additional resources that might help people to adopt the healthy behaviors recommended on the MYM website were suggested, and respondents were asked to rate how helpful these would be to them. The high ratings given suggested that respondents’ motivation to address their health related to dementia risk was high, and that in general people were looking for practical resources to assist them. Personalized assessments of their risk factors, tailored advice to address risk factors, and assessment results to take to their doctor were rated highest. Previous studies have similarly found that health website users would like to see more interactivity and more specific practical information that tells them exactly what they need to do, making it easy for them to adopt healthier behaviors [26].

Survey respondents were predominantly female and well-educated, consistent with findings that these demographic characteristics were associated with more frequent health-related Internet use [25,27,37,38]. Respondents were also predominantly older, with 86/122 (70.5%) aged over 50 and 55/122 (45.1%) aged over 60. This was likely related to community survey findings which suggested that more people over age 50 worry about developing dementia [33], and to the most selected reasons for interest in the MYM website being related to concerns about dementia. Nevertheless, this self-selected sample limits the generalizability of the findings to other sectors of the community, younger people and males in particular. Perhaps future research and development of Web-based health resources could address promotion strategies aimed specifically at men [27,39].

Respondents rated their current healthy behavior related to the risk or protective factors on average as doing “pretty well”, suggesting the people most likely to access the MYM website and respond to the survey felt that they were already leading a healthy life. This was consistent with previous findings and suggests that another important target group for future Web-based health interventions is those with weak health motivation [27,39]. While people who are more committed to a healthy lifestyle are more likely to seek and use Web-based health information, it is important to reach those who could benefit the most from these Internet interventions, that is, those who engage in unhealthy risk behaviors. However, as people will pursue information sources in relation to their own interests and needs, this is not straightforward.

### Limitations and Future Studies

Generalizability of the findings from the current study to the general community was limited by the use of a self-selected sample, and a lack of data on visitors to the website who did not complete the survey. Because an opt-in survey was used, it was not possible to calculate a response rate for the survey or to determine any demographic differences between survey respondents and visitors who did not participate in the survey. While the resulting sample size was modest, the study was able to detect significant positive effects of visiting the MYM website.

A further limitation of the current study was that knowledge about risk or protective factors for dementia and the importance ascribed to them were not assessed before participants viewed the website. Their subjective report of prior knowledge and importance were assessed with the survey after they had read the website. The study was also unable to measure whether participants actually made lifestyle changes. Nevertheless, respondents’ felt their knowledge had increased, as had the importance they ascribed to monitoring their health-related behavior.

Further longitudinal studies are required to better assess knowledge prior to exposure to the MYM information, and any lifestyle modifications after exposure. Future research should also examine whether the beneficial effects of providing dementia risk reduction information can be enhanced by also providing interactive and personalized Web-based features. Enhancements to the MYM website are in development which will include the ability for users to assess their dementia risk and be provided with tailored advice and resources. Research is also underway to evaluate whether such an interactive program results in better health outcomes for users than an information website alone. This ongoing research program aims to inform future developments of dementia prevention initiatives for Australia [9].

### Conclusions

This study has demonstrated that a dementia risk reduction website successfully increased users’ knowledge and the importance they attributed to monitoring their health behavior. Positive ratings of the website content suggest it was relevant to users and presented in a manner they easily understood. Users felt well-equipped to improve their behavior and found the practical tips provided useful. They also felt that suggested additional resources would be helpful, such as interactive and personalized resources on the website to further engage people to enable behavior change. These findings will inform future developments of the MYM program and website.

## Abbreviations

MYM: Mind your Mind
